# Decoding the Nonlinear Association Between Visceral Adiposity Index and All‐Cause Mortality: The Mediating Role of White Blood Cells and Neutrophils

**DOI:** 10.1155/ije/3116986

**Published:** 2025-12-30

**Authors:** Yanmei Yu, Tongcai Tan, Wei Yang, Zhitao Xu, Yong Liu

**Affiliations:** ^1^ Center for Rehabilitation Medicine, Rehabilitation & Sports Medicine Research Institute of Zhejiang Province, Department of Rehabilitation Medicine, Zhejiang Provincial People’s Hospital(Affiliated People’s Hospital, Hangzhou Medical College), Hangzhou, Zhejiang, China

**Keywords:** all-cause mortality, inflammation, mediation analysis, NHANES, obesity, visceral adiposity index

## Abstract

**Background:**

The visceral adiposity index (VAI) is recognized as a crucial metabolic risk indicator associated with mortality and is widely used in clinical and epidemiological studies. Evidence suggests that systemic inflammation may mediate this link; however, the underlying mechanisms remain poorly understood. To advance our understanding of these critical health risks, additional studies are required to clarify the underlying pathways connecting VAI, inflammation, and mortality.

**Methods:**

Using data from the National Health and Nutrition Examination Survey (NHANES), to investigate the link between log_2_‐VAI and all‐cause mortality. To evaluate correlations, this study applied Kaplan–Meier survival curves alongside Cox proportional hazards models, and potential nonlinear associations were analyzed through the application of restricted cubic spline (RCS) models. The consistency of associations in populations with different demographic and clinical characteristics was assessed by subgroup analyses, and the role of inflammatory markers was investigated by mediation analyses.

**Results:**

This study highlighted a nonlinear connection between log_2_‐VAI and all‐cause mortality, pinpointing a risk threshold at log_2_‐VAI = 1.81. Beyond this threshold, the likelihood of all‐cause mortality increased significantly. Subgroup analyses identified a notably stronger association among middle‐aged groups (40–60 years) and those without coronary heart disease (CHD). Mediation analysis demonstrated that systemic inflammatory markers, specifically white blood cell count (WBC) and neutrophil count, mediated 45.07% and 37.91% of the association, respectively. E‐value analysis suggested robustness to unmeasured confounding.

**Conclusion:**

This study underscores the importance of WBC and neutrophil counts as key mediators linking VAI to all‐cause mortality, offering fresh perspectives on the metabolic and inflammatory pathways underlying this association. These results highlight the critical role of public health interventions targeting inflammation to mitigate obesity‐related mortality risks.

## 1. Introduction

As reported by the World Health Organization (WHO), the global prevalence of obesity is projected to reach 18% by 2025, imposing a substantial burden on healthcare systems and economies worldwide [1]. Obesity is a major contributor to chronic conditions and significantly elevates all‐cause mortality [2]. While the body mass index (BMI) is widely used to assess obesity [3], its predictive ability for health risks remains limited due to its inability to capture visceral fat distribution and metabolic function [4].

In recent years, the visceral adiposity index (VAI) has gained recognition as a comprehensive indicator that integrates variables such as sex, waist circumference (WC), body weight, and lipid levels to more sensitively assess obesity‐related metabolic risks [5]. VAI levels also vary with age, reflecting age‐related shifts in fat distribution and lipid metabolism [6]. Compared to BMI, VAI offers a precise representation of abdominal fat distribution and metabolic function [7]. It has demonstrated strong predictive capabilities for all‐cause mortality in numerous studies, making it a valuable supplement to the traditional BMI [6, 8, 9].

Previous studies have indicated a notable link between VAI and all‐cause mortality, with strong predictive capabilities observed in older adults and dialysis patients [5, 10]. Nonetheless, prior research is constrained by sample biases and the absence of comprehensive analyses in general adult populations. Additionally, existing literature predominantly focuses on the epidemiological aspects of VAI’s predictive role, with limited attention given to its underlying biological mechanisms [9, 11]. In particular, inflammation, a critical mediator of obesity‐related health risks, has not been adequately validated or investigated about VAI and all‐cause mortality.

To address these research gaps, this study analyzed data from a United States (US) cohort surveyed in the National Health and Nutrition Examination Survey (NHANES) program between 1999 and 2018. It systematically explored the link between VAI and all‐cause mortality, comprehensively evaluated its applicability across different subgroups, and, for the first time, quantified the risk threshold of VAI. Furthermore, the study investigated the mediating role of systemic inflammation in this relationship by examining key inflammatory biomarkers, including white blood cell count (WBC), neutrophil count, lymphocyte count, red blood cell distribution width (RDW), and the neutrophil‐to‐lymphocyte ratio (NLR), providing novel insights into the biological pathways linking metabolic risk to mortality.

## 2. Materials and Methods

### 2.1. Study Design and Population

**Figure 1:**
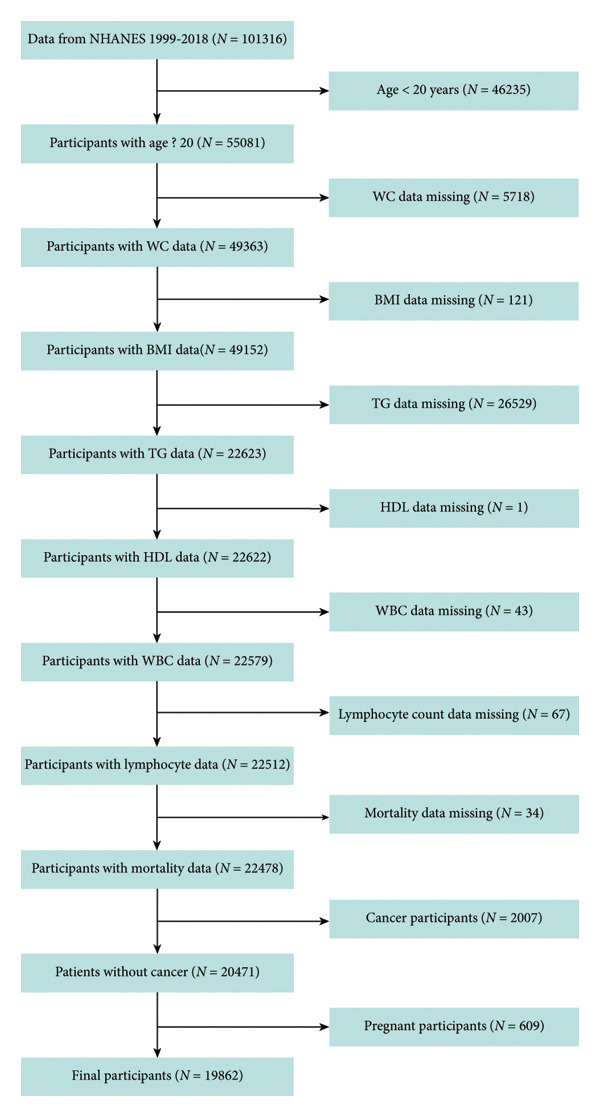
The flowchart of participant selection. NHANES, National Health and Nutrition Examination Survey; WC, waist circumference; BMI, body mass index; TG, triglyceride; HDL‐C, high‐density lipoprotein cholesterol; WBC, white blood cell.

This study utilizes NHANES data and follows a longitudinal cohort design to ensure a comprehensive analysis over time. It uses multistage probability sampling to ensure national representativeness, with data collected through interviews, clinical assessments, and laboratory evaluations [12]. Strict oversight was applied throughout data collection and processing, and all procedures were conducted by professionally trained healthcare personnel. NHANES cycles (1999–2018) provided the dataset used in this study. An aggregate of 46,235 individuals under 20 years of age was excluded, along with 5718 participants without WC data, 211 participants without BMI data, 26,529 participants without triglyceride (TG) data, 1 participant without high‐density lipoprotein cholesterol (HDL‐C) data, 43 participants without WBC data, 67 participants without lymphocyte count data, 34 participants without mortality data, 2007 individuals with prior cancer diagnoses, and 609 pregnant women. Ultimately, the analysis included 19,862 participants. The process for selecting the sample is shown in Figure 1.

### 2.2. Assessment of VAI

The VAI is a validated marker of visceral fat distribution and metabolic risk, integrating WC, BMI, TG, and HDL‐C. VAI is calculated using a sex‐specific formula, where TG and HDL‐C are measured in millimoles per liter (mmol/L), BMI in kilograms per square meter (kg/m^2^), and WC in centimeters (cm). This index provides a functional assessment of visceral adiposity and has been widely utilized as a predictor of cardiovascular risk, insulin resistance, and all‐cause mortality. Because VAI is sex‐specific and population‐dependent, a universally accepted “normal” cut‐off is not available. In the original derivation, healthy, nonobese individuals with normal adipose distribution and normal TG/HDL levels were calibrated to VAI ≈ 1 [13]. For this cohort, we categorized VAI into sex‐specific quintiles using cohort‐specific cut points (see Supporting Table S2). The VAI was calculated using the following formula [14]:

(1) For Men:VAI=WC39.681.88+×BMI×TG1.03+1.31HDL−C,

(2) For Women:VAI=WC36.581.89+×BMI×TG0.81+1.52HDL−C.

### 2.3. Assessment of All‐Cause Mortality

This study focused on all‐cause mortality as the primary outcome. Mortality status was determined using publicly available NHANES‐linked mortality files [15] (updated through December 31, 2019) and the National Death Index (NDI) [16]. These data were provided by the National Center for Health Statistics (NCHS) through a probabilistic matching algorithm. Additional details are available through the official resources of the Centers for Disease Control and Prevention (CDC).

### 2.4. Covariables

The study collected covariates including demographic factors (age, sex, race, education level, and poverty‐to‐income ratio); lifestyle behaviors (marital status, smoking history, alcohol intake, and physical activity); and clinical measures. The clinical measures included cardiovascular conditions (congestive heart failure [CHF], coronary heart disease [CHD], and stroke), chronic illnesses (hypertension, hyperlipidemia, and diabetes), anthropometric indices (BMI and WC), and biochemical parameters (HDL‐C, TG, low‐density lipoprotein cholesterol [LDL‐C], and total cholesterol [TC]). Additionally, hematological markers such as RDW, WBC, lymphocyte count, neutrophil count, and NLR were included.

### 2.5. Statistical Analysis

NHANES employed a multistage sampling framework, and statistical analyses incorporated weights to produce nationally representative findings. Statistical analyses were carried out with R (version 4.2.2) and EmpowerStats (version 4.2). Since VAI exhibited a skewed distribution, it was log_2_‐transformed prior to statistical analysis. Additionally, log_2_‐VAI was categorized into quartile‐based groups after being transformed from a continuous variable. Frequencies and percentages were used to summarize categorical variables, and group comparisons were conducted via the chi‐square test. Weighted averages and their corresponding 95% confidence intervals (CIs) were used to represent continuous variables, and group differences were evaluated using weighted linear regression analysis. Statistical significance in all analyses was determined using a threshold of *p*  <  0.05.

Survival probabilities among VAI quartiles were evaluated using Kaplan–Meier analysis. To assess group differences, the log‐rank test was employed. Cox proportional hazards models were employed to analyze the link between log_2_‐transformed VAI and all‐cause mortality, estimating hazard ratios (HRs) and 95% CI for both log_2_‐VAI (continuous) and VAI quartiles (categorical). Three distinct models were proposed: The first was without covariate adjustments. Adjustments for age, sex, and race were applied in the second model. The third model included additional adjustments for socioeconomic factors (education level, poverty‐to‐income ratio, and marital status), lifestyle variables (smoking, alcohol consumption, and physical activity), cardiovascular conditions (CHF, CHD, and stroke), hematological markers (WBC, lymphocyte count, neutrophil count, RDW, and NLR), and metabolic conditions (hypertension, diabetes, and TC).

To analyze the nonlinear correlation between log_2_‐VAI and all‐cause mortality, restricted cubic spline (RCS) regression was employed, with covariates adjusted throughout the models. Probability trends were assessed using 95% CI. A segmented regression model with a log‐likelihood comparison test was employed to explore the potential threshold effect of log_2_‐VAI on all‐cause mortality, with relevant confounders adjusted.

Additionally, subgroup analyses were utilized to examine the link between VAI and all‐cause mortality across categories, including demographics (sex, age, race, and marital status), socioeconomic factors (education and income), behaviors (smoking and physical activity), and health conditions (CHF, CHD, stroke, hypertension, and diabetes). This study employed causal mediation analysis to evaluate the mediating effects of WBC, neutrophil count, lymphocyte count, RDW, and NLR on the association between log_2_‐VAI and all‐cause mortality. The analysis was conducted using EmpowerStats, with indirect effects (IEs) estimated via a nonparametric bootstrap resampling approach (X resamples) to generate bias‐corrected 95% CI. The total effect of log_2_‐VAI on all‐cause mortality was decomposed into direct effects (DEs) and IEs, with the mediation proportion calculated accordingly. A mediation effect was considered statistically significant if the 95% CI of the IE did not include zero.

To assess the robustness of our findings to unmeasured confounding, we calculated the *E*‐value using EmpowerStats. This analysis quantifies the minimum strength of association that an unmeasured confounder would need to have with both log_2_‐VAI and all‐cause mortality to explain away the observed relationship fully [17].

## 3. Results

### 3.1. Baseline Characteristics

Table 1 presents the weighted sociodemographic and covariate distributions stratified by log_2_‐VAI quartiles. This study involved 19,862 participants, with an average age of 48.54 years. Of these, 50.13% identified as male, while 42.19% identified as non‐Hispanic White participants. The mean VAI was 4.85, and the mean log_2_‐VAI was 1.81. Log_2_‐VAI was divided into the following quartiles: (1) < 1.04; (2) 1.04–1.76; (3) 1.76–2.52; and (4) > 2.52. Participants in higher log_2_‐VAI quartiles tended to be older, predominantly male, more frequently non‐Hispanic White, and more likely to have higher education and moderate poverty‐to‐income ratios. They were also more often married or cohabiting with a partner. Additionally, these participants showed greater smoking history prevalence, reduced physical activity levels, and greater BMI and WC. In terms of disease prevalence, participants in higher log_2_‐VAI quartiles showed a significantly greater proportion of hypertension, diabetes, stroke, CHF, and CHD. Regarding biochemical markers, these participants had lower HDL‐C levels but significantly higher levels of TGs, LDL‐C, TC, and hematological counts (WBC, neutrophils, lymphocytes, and NLR).

**Table 1:** Characteristics of participants in the NHANES 1999–2018 cycle.

Characteristics	Visceral adiposity index				*p* value
	Q1	Q2	Q3	Q4	
	(< 1.04)	(1.04–1.76)	(1.76–2.52)	(> 2.52)	
Participants	*N* = 4966	*N* = 4965	*N* = 4965	*N* = 4966	
Age (years)	42.53 (41.81, 43.25)	45.29 (44.65, 45.93)	47.06 (46.48, 47.65)	48.62 (48.08, 49.16)	< 0.0001
Gender (%)					< 0.001
Male	2636 (53.08%)	2432 (48.98%)	2388 (48.10%)	2500 (50.34%)	
Female	2330 (46.92%)	2533 (51.02%)	2577 (51.90%)	2466 (49.66%)	
Race/ethnicity (%)					< 0.001
Non‐Hispanic White	1852 (37.29%)	2093 (42.16%)	2109 (42.48%)	2326 (46.84%)	
Non‐Hispanic Black	1576 (31.74%)	1159 (23.34%)	834 (16.80%)	520 (10.47%)	
Mexican American	623 (12.55%)	802 (16.15%)	1072 (21.59%)	1223 (24.63%)	
Other races	915 (18.43%)	911 (18.35%)	950 (19.13%)	897 (18.06%)	
Education level (%)					< 0.001
< High school	1049 (21.14%)	1239 (24.97%)	1497 (30.21%)	1689 (34.07%)	
High school	1049 (21.14%)	1145 (23.08%)	1127 (22.74%)	1211 (24.43%)	
> High school	2864 (57.72%)	2577 (51.95%)	2332 (47.05%)	2058 (41.51%)	
Income‐to‐poverty ratio (%)					< 0.001
< 1.5	1454 (29.28%)	1535 (30.92%)	1693 (34.10%)	1856 (37.37%)	
1.5–3.5	1935 (38.96%)	1930 (38.87%)	1972 (39.72%)	1875 (37.76%)	
> 3.5	1577 (31.76%)	1500 (30.21%)	1300 (26.18%)	1235 (24.87%)	
Marital status (%)					< 0.001
Married/living with partner	2783 (56.39%)	2964 (60.16%)	3085 (62.83%)	3159 (64.48%)	
Widowed/divorced/separated	866 (17.55%)	1032 (20.95%)	1082 (22.04%)	1131 (23.09%)	
Never married	1286 (26.06%)	931 (18.90%)	743 (15.13%)	609 (12.43%)	
Smoked at least 100 cigarettes (%)					< 0.001
Yes	1982 (39.98%)	2179 (43.91%)	2301 (46.37%)	2588 (52.16%)	
No	2976 (60.02%)	2783 (56.09%)	2661 (53.63%)	2374 (47.84%)	
Physical activity (%)					< 0.001
Yes	2119 (42.69%)	2030 (40.92%)	2013 (40.57%)	1905 (38.38%)	
No	2845 (57.31%)	2931 (59.08%)	2949 (59.43%)	3059 (61.62%)	
CHF (%)					< 0.001
Yes	80 (1.61%)	107 (2.16%)	138 (2.79%)	221 (4.46%)	
No	4877 (98.39%)	4845 (97.84%)	4811 (97.21%)	4729 (95.54%)	
CHD (%)					< 0.001
Yes	116 (2.34%)	150 (3.03%)	203 (4.11%)	246 (4.99%)	
No	4840 (97.66%)	4793 (96.97%)	4738 (95.89%)	4688 (95.01%)	
Stroke (%)					< 0.001
Yes	105 (2.12%)	152 (3.06%)	163 (3.29%)	198 (3.99%)	
No	4858 (97.88%)	4808 (96.94%)	4797 (96.71%)	4761 (96.01%)	
Hypertension (%)					< 0.001
Yes	676 (13.98%)	832 (17.27%)	948 (19.76%)	1103 (22.89%)	
No	4158 (86.02%)	3986 (82.73%)	3850 (80.24%)	3716 (77.11%)	
Diabetes (%)					< 0.001
Yes	257 (5.18%)	392 (7.91%)	645 (13.03%)	1046 (21.13%)	
No	4705 (94.82%)	4563 (92.09%)	4307 (86.97%)	3905 (78.87%)	
Hyperlipidemia (%)					< 0.001
Yes	1735 (34.94%)	2662 (53.62%)	3959 (79.74%)	4956 (99.80%)	
No	3231 (65.06%)	2303 (46.38%)	1006 (20.26%)	10 (0.20%)	
BMI (%)					< 0.001
< 25	2582 (51.99%)	1682 (33.88%)	1047 (21.09%)	665 (13.39%)	
25–30	1438 (28.96%)	1742 (35.09%)	1788 (36.01%)	1806 (36.37%)	
≥ 30	946 (19.05%)	1541 (31.04%)	2130 (42.90%)	2495 (50.24%)	
BMI (kg/m^2^)	25.54 (25.31, 25.77)	27.93 (27.65, 28.20)	29.99 (29.74, 30.25)	31.39 (31.14, 31.64)	< 0.0001
WC (cm)	89.09 (88.51, 89.67)	95.88 (95.22, 96.53)	101.74 (101.15,102.33)	106.30 (105.65,106.94)	< 0.0001
HDL‐C (mg/dL)	67.36 (66.68, 68.05)	56.05 (55.57, 56.53)	48.93 (48.57, 49.28)	40.58 (40.22, 40.94)	< 0.0001
TG (mg/dL)	59.05 (58.41, 59.69)	91.23 (90.42, 92.04)	129.26 (128.02,130.51)	247.76 (241.77,253.75)	< 0.0001
LDL‐C (mg/dL)	103.64 (102.47,104.80)	116.18 (114.97,117.39)	122.89 (121.46,124.31)	121.45 (120.13,122.77)	< 0.0001
TC (mg/dL)	182.70 (181.33,184.07)	190.46 (189.03,191.88)	197.81 (196.17,199.45)	209.53 (207.81,211.25)	< 0.0001
Alcohol use	2.22 (2.14, 2.30)	2.24 (2.15, 2.32)	2.21 (2.13, 2.29)	2.25 (2.16, 2.35)	0.9022
RDW (%)	13.02 (12.98,13.06)	12.99 (12.94,13.04)	12.96 (12.91,13.00)	12.97 (12.92,13.01)	0.1462
WBC (×10^9^/L)	6.09 (6.02,6.16)	6.65 (6.56,6.73)	6.98 (6.91,7.05)	7.44 (7.35,7.53)	< 0.0001
Lymphocyte count (×10^9^/L)	1.84 (1.82,1.87)	1.95 (1.92,1.97)	2.06 (2.03,2.08)	2.18 (2.15,2.21)	< 0.0001
Neutrophil count (×10^9^/L)	3.52 (3.46,3.57)	3.92 (3.86,3.98)	4.13 (4.08,4.18)	4.42 (4.35,4.49)	< 0.0001
NLR	2.06 (2.02,2.10)	2.17 (2.13,2.21)	2.16 (2.12,2.20)	2.18 (2.14,2.22)	0.0002

*Note:* Categorical variables were expressed as frequencies and percentages, and intergroup differences were analyzed using the weighted chi‐square test. Continuous variables were expressed as weighted means and 95% CI, and intergroup differences were assessed using the weighted linear regression model. RDW, red cell distribution width; TG, triglyceride; WBC, white blood cell count.

Abbreviations: BMI, body mass index; CHD, coronary heart disease; CHF, congestive heart failure; HDL‐C, high‐density lipoprotein cholesterol; LDL‐C, low‐density lipoprotein cholesterol; NLR, neutrophil‐to‐lymphocyte ratio; TC, total cholesterol; WC, waist circumference.

### 3.2. Association Between log_2_‐VAI and Mortality

**Figure 2:**
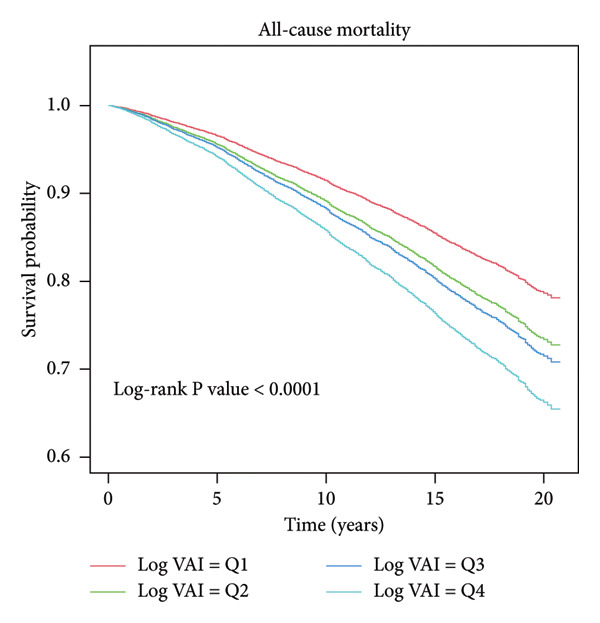
Kaplan–Meier curves for all‐cause mortality in four groups.

Throughout the follow‐up period, a median of 118.3 months (covering 1–249 months), a total of 2622 deaths were recorded, accounting for 13.2% of all participants. Kaplan–Meier survival analysis revealed that higher log_2_‐VAI quartiles were strongly linked to elevated all‐cause mortality and reduced survival rates. During the observation period, individuals in Q4 exhibited the highest cumulative incidence of all‐cause mortality relative to those in Q1 (log‐rank *p*  <  0.001, Figure 2). The link between log_2_‐VAI and all‐cause mortality is given in Table 2. According to the crude model, each unit increase in log_2_‐VAI corresponded to a 24% higher hazard of all‐cause mortality (HR: 1.24, 95% CI: 1.19–1.30, *p*  <  0.001). According to the partially adjusted model, the link was marginally weaker but remained statistically significant (HR: 1.16, 95% CI: 1.10–1.22). In the model adjusted for all confounders, each unit increase in log_2_‐VAI corresponded to an 8% higher hazard of all‐cause mortality (HR: 1.08, 95% CI: 1.02–1.14, *p*  <  0.001). Additionally, when log_2_‐VAI was grouped into quartiles, individuals in Q4 had a significantly greater risk of all‐cause mortality relative to those in Q1 (fully adjusted HR: 1.22, 95% CI: 1.05–1.43, *p* for trend < 0.001).

**Table 2:** Association between the log_2_‐VAI and all‐cause mortality.

	Crude	Partially	Fully
	**Model**	**Adjusted model**	**Adjusted model**
	**(Model 1)**	**(Model 2)**	**(Model 3)**
	**HR (95% CI)**	**HR (95% CI)**	**HR (95% CI)**
log_2_‐VAI	1.24 (1.19, 1.30)	1.16 (1.1, 1.22)	1.08 (1.02, 1.14)
log_2_‐VAI quartile			
Quartile 1 (< 1.04)	Reference	Reference	Reference
Quartile 2 (1.04–1.76)	1.33 (1.13, 1.56)	1.06 (0.89, 1.26)	1.01 (0.84, 1.22)
Quartile 3 (1.76–2.52)	1.49 (1.26, 1.76)	1.08 (0.91, 1.26)	0.98 (0.82, 1.16)
Quartile 4 (> 2.52)	2.07 (1.77, 2.42)	1.46 (1.25, 1.70)	1.22 (1.05, 1.43)
*p* for trend	< 0.001	< 0.001	< 0.001

*Note:* The bold text is used to highlight the column headings representing different regression models and outcome measures (i.e., Model 1, Model 2, Model 3, and HR [95% CI]). Model 1: no covariates were adjusted. Model 2: age, gender, and race were adjusted. Model 3: age, gender, race, education level, income‐to‐poverty ratio, marital status, smoking status, alcohol use, physical activity, CHF, CHD, stroke, WBC, lymphocyte count, neutrophil count, RDW, NLR, hypertension, diabetes, and TC were adjusted. VAI, visceral fat index; WBC, white blood cell count; RDW, red blood cell distribution width.

Abbreviations: CHD, coronary heart disease; CHF, congestive heart failure; NLR, neutrophil‐to‐lymphocyte ratio; TC, total cholesterol.

### 3.3. Nonlinear Association Between log_2_‐VAI and Mortality

**Figure 3:**
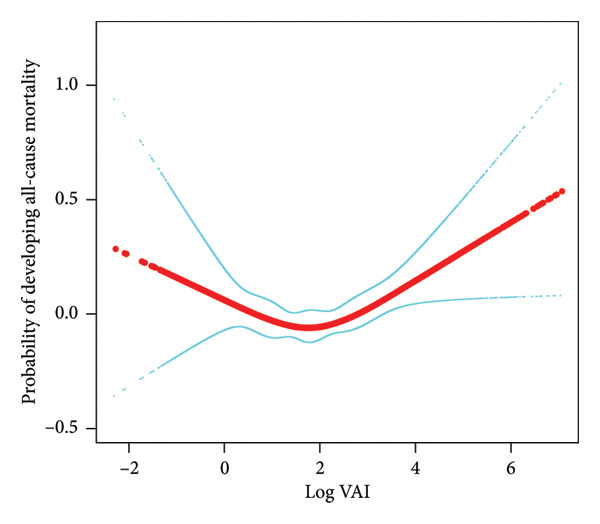
The nonlinear associations between log_**2**_ ‐VAI and all‐cause mortality. The solid red line represents the estimated probability of all‐cause mortality based on a RCS model, adjusted for covariates. The light blue lines denote the 95% CI. The *x*‐axis corresponds to log_2_‐VAI, while the *y*‐axis represents the probability of all‐cause mortality.

A nonlinear correlation existed between log_2_‐VAI and all‐cause mortality was shown by smoothing curve fitting and threshold effect analysis (Figure 3). As log_2_‐VAI levels increased, the mortality risk initially decreased and then increased, with a turning point observed at log_2_‐VAI = 1.81, as shown in Table 3. Below this threshold, the association was not significant (HR: 0.93, 95% CI: 0.85–1.02, *p* = 0.1183). Above the threshold, the hazard of all‐cause mortality increased significantly (HR: 1.12, 95% CI: 1.05–1.20, *p* = 0.0004). The likelihood ratio test confirmed a significant threshold effect (*p*  <  0.005). These results accounted for various confounders, such as demographic traits, lifestyle habits, and comorbidities.

**Table 3:** Threshold analysis for the relationship between log_2_‐VAI and all‐cause mortality.

Models	Adjusted HR (95% CI); *p* value
Model I	
One line slope	1.05 (1.00, 1.09); 0.0400
Model II	
Turning point (K)	1.81
< 1.81	0.93 (0.85, 1.02); 0.1183
> 1.81	1.12 (1.05, 1.20); 0.0004
HR between < 1.81 and > 1.81	1.21 (1.06, 1.37); 0.0041
Logarithmic likelihood ratio test	< 0.005

*Note:* Adjusted for: sex, age, race, education, income‐to‐poverty ratio, marital status, smoking status, alcohol use, physical activity, CHF, CHD, stroke, WBC, lymphocyte count, neutrophil count, RDW, NLR, hypertension, diabetes, and TC. VAI, visceral fat index; WBC, white blood cell count; RDW, red blood cell distribution width.

Abbreviations: CHD, coronary heart disease; CHF, congestive heart failure; NLR, neutrophil‐to‐lymphocyte ratio; TC, total cholesterol.

### 3.4. Subgroup Analyses

**Figure 4:**
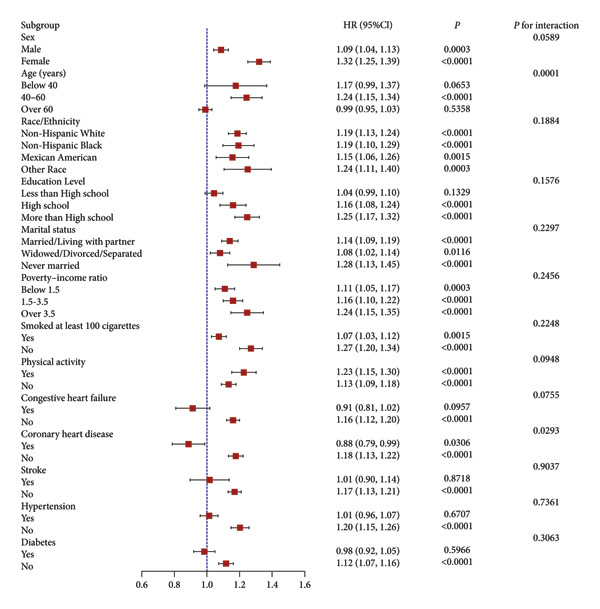
Subgroup analysis of the association between log_2_‐VAI and all‐cause mortality.

To evaluate the stability of the link between log_2_‐VAI and all‐cause mortality across different cohorts and to identify potential population differences, analyses were stratified by sex, age, race, education, marital status, income level, smoking status, physical activity, CHF, CHD, stroke, hypertension, and diabetes through subgroup and interaction tests. As shown in Figure 4, the association differed significantly across certain subgroups. In age‐focused analysis, log_2_‐VAI was significantly linked with all‐cause mortality in individuals aged 40–60 years (HR: 1.24, 95% CI: 1.15–1.34; *p*  <  0.0001), but not among those aged < 40 years (HR: 1.17, 95% CI: 0.99–1.37) or > 60 years (HR: 0.99, 95% CI: 0.95–1.03) (*p* for interaction = 0.0001). Additionally, within the CHD subgroup analysis, log_2_‐VAI was strongly linked to all‐cause mortality in participants without CHD (HR: 1.18, 95% CI: 1.13–1.22; *p*  <  0.0001), whereas the association was not statistically significant in participants with CHD (HR: 0.88, 95% CI: 0.79–0.99; *p* = 0.0306, *p* for interaction = 0.0293). In other subgroups, although the connection between log_2_‐VAI and all‐cause mortality exhibited significant differences, none of the evaluated interactions showed significant results (*p* for interaction > 0.05).

### 3.5. The Mediating Role of Inflammation‐Related Indicators

**Figure 5:**
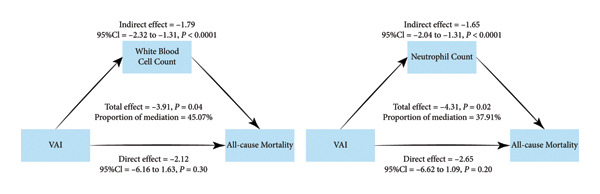
The mediating effects of WBC and neutrophil count on the relationship between log_2_‐VAI and all‐cause mortality.

Figure 5 illustrates that after adjusting for confounders, WBC mediated 45.07% of the association between log_2_‐VAI and all‐cause mortality, with a significant IE of −1.79 (95% CI: −2.32 to −1.31, *p*  <  0.0001). For neutrophil count, the mediation proportion was 37.91%, with an IE of −1.65 (95% CI: −2.04 to −1.31, *p*  <  0.0001). In both models, the DE of log_2_‐VAI on all‐cause mortality was not statistically significant (*p*  >  0.05). Additionally, mediation analyses of other inflammatory biomarkers, including lymphocyte count, RDW, and NLR, showed no substantial mediation effects, as summarized in Supporting Table S1.

### 3.6. Sensitivity Analysis

We calculated the *E*‐value using EmpowerStats to evaluate the potential impact of unmeasured confounders on the association between VAI and all‐cause mortality. For the primary analysis (HR = 0.72), the *E*‐value was 2.12, indicating that an unmeasured confounder would need to be associated with both VAI and all‐cause mortality by a risk ratio of at least 2.12 to explain away the observed association completely. Furthermore, if the confounder’s association with VAI is assumed to be 1.67, its association with mortality would need to reach an RR of ≥ 3.31 to nullify the observed effect. Therefore, unless there is an extremely strong unmeasured confounder, our results are considered robust.

## 4. Discussion

This study identified a strong link between log_2_‐VAI and all‐cause mortality and, for the first time, clarified the nonlinear characteristics of this relationship, with log_2_‐VAI = 1.81 identified as the risk turning point. The results demonstrated a significant link between each unit increment in log_2_‐VAI and elevated all‐cause mortality risk, particularly among middle‐aged individuals (40–60 years) and those without CHD. Additionally, mediation analysis systematically demonstrated for the first time that WBC and neutrophil counts mediated 45.07% and 37.91% of the link between log_2_‐VAI and all‐cause mortality, respectively, indicating that inflammation is a central mechanism underlying this relationship. Previous studies have extensively explored the association between the VAI and mortality, confirming its potential as a significant predictor of metabolic risk and survival outcomes. However, these investigations often suffered from limitations such as restricted study populations, relatively short follow‐up durations, and inadequate exploration of potential biological mechanisms. For instance, an analysis based on NHANES 2011–2014 demonstrated that age‐adjusted VAI independently predicted all‐cause and cardiovascular mortality [6]; however, its limited sample size and short follow‐up period hindered long‐term inference. Zhang et al. [18] examined adults aged 20–65 years and found no significant nonlinear association between VAI and all‐cause mortality, although a threshold of 2.49 was identified for cardiovascular mortality; nevertheless, the age‐limited cohort and outcome‐specific focus restricted the generalizability of their findings. Yan et al. [11] reported a J‐shaped relationship between VAI and all‐cause mortality in patients with chronic kidney disease, but the extremely high inflection point (VAI ≈ 68.23) and disease‐specific sample limited its clinical applicability. Similarly, Luo et al. [8] revealed a nonlinear association between VAI and CHD, with higher VAI levels linked to increased CHD risk and poorer survival outcomes, yet the analysis was confined to a disease‐specific endpoint. Sun et al. [19]identified a U‐shaped association between VAI and all‐cause mortality in a general adult population, with a turning point at log_2_‐VAI = 0.824 and a stronger effect among younger individuals; however, the study primarily emphasized curve shape and age interactions, without determining a clinically interpretable threshold or exploring potential biological pathways. Furthermore, previous studies, such as those based on the United Kingdom Biobank (UK Biobank), have validated the positive link between VAI and all‐cause mortality using extensive samples and prolonged follow‐up periods [10], which aligns with the findings of this study. However, these analyses primarily remain at the descriptive epidemiological level, without quantifying specific risk thresholds or exploring potential mechanisms in depth. Similarly, certain studies have proposed that VAI could be linked to metabolic disorders and inflammation [20–22], but none have systematically analyzed the underlying mechanisms.

In contrast, the present study, based on nationally representative NHANES data from 1999 to 2018, overcomes the aforementioned limitations. With a follow‐up period of nearly 20 years, the study verified the nonlinear association between VAI and all‐cause mortality using RCS and two‐piecewise Cox regression models and further confirmed the robustness of the results through E‐value sensitivity analysis. These methodological strengths enhance both the statistical reliability and mechanistic interpretability of the findings, providing greater external validity and clinical relevance. For the first time, this study identified a clinically meaningful threshold for all‐cause mortality (log_2_‐VAI = 1.81, approximately 3.5) in a general adult population and demonstrated that systemic inflammatory markers—WBC and neutrophil count—partially mediated the association between VAI and mortality, thereby uncovering novel biological insights into the underlying mechanisms. To further elucidate the role of inflammation in this relationship, multiple inflammatory biomarkers—including C‐reactive protein (CRP), interleukin‐6 (IL‐6), tumor necrosis factor‐alpha (TNF‐α), WBC, neutrophil count, RDW, and lymphocyte count—were initially considered as potential mediators. However, IL‐6 and TNF‐α were unavailable in the NHANES dataset during the study period, and CRP data were incomplete, precluding their inclusion in the analysis. To ensure a comprehensive evaluation, WBC, neutrophil count, RDW, and lymphocyte count were incorporated into the mediation analysis model to systematically assess their roles in the association between VAI and mortality. The results revealed that WBC and neutrophil count mediated 45.07% and 37.91% of the association, respectively, suggesting that these inflammatory markers may act as key biological regulators linking VAI to all‐cause mortality by promoting inflammatory activation and metabolic dysfunction.

As a hallmark of obesity‐associated metabolic disorders [23], chronic inflammation serves as a critical mediator linking obesity to increased mortality risk [24]. Earlier research has highlighted inflammation as a significant contributor to obesity‐related mortality. For example, the study demonstrated that the inflammatory state reflected by SII significantly predicted long‐term mortality risk [25]. Additionally, the study further confirmed that neutrophils play a crucial role in cardiovascular mortality by inducing vascular inflammatory responses. In obese individuals, the number of pro‐inflammatory M1 macrophages and Th1 cells in adipose tissue is significantly increased [26, 27], whereas regulatory T cells (Tregs) are reduced [28, 29]. The imbalance in immune cells contributes to the persistent secretion of pro‐inflammatory cytokines, such as MCP‐1, IL‐6, and TNF‐α, which induces insulin resistance and dyslipidemia [30, 31]. These metabolic abnormalities further activate neutrophils and WBCs through circulating inflammatory factors, perpetuating systemic inflammation [32, 33].

The pro‐inflammatory mechanisms of neutrophils are particularly critical in this process [34]. Neutrophils directly damage vascular endothelium by releasing reactive oxygen species (ROS) and elastase [35], while also promoting atherosclerosis and tissue injury through the enhancement of oxidative stress [36]. A rise in WBC, a broad marker of systemic inflammation, reflects heightened immune activation [37]. This elevation is not only associated with increased levels of oxidative stress but also exacerbates metabolic disorders by contributing to vascular remodeling and immune dysfunction [38]. These mechanisms significantly increase the risk of all‐cause mortality.

Intervention studies have demonstrated that regulating inflammation can reduce mortality risk in individuals with high VAI. For instance, the Mediterranean diet significantly decreases metabolic risk and associated inflammatory levels of VAI by reducing adipose tissue inflammation [39]. Additionally, pharmacological interventions like metformin have been shown to regulate macrophage polarization and inhibit the pro‐inflammatory activity of neutrophils by activating the AMPK signaling pathway [40], further supporting the feasibility of reducing mortality risk through inflammation modulation.

This study first quantitatively identified log_2_‐VAI = 1.81 as the nonlinear threshold for mortality risk. When log_2_‐VAI was below this value, mortality risk remained relatively low, whereas exceeding 1.81 was associated with a significant increase in mortality risk. This nonlinear relationship likely reflects a transition from metabolic homeostasis to metabolic dysfunction in adipose tissue. At log_2_‐VAI < 1.81, adipose tissue retains a sufficient lipid storage capacity, effectively buffering free fatty acids (FFAs) and preventing their excessive deposition in the liver, pancreas, and vasculature, thereby reducing lipotoxicity and insulin resistance [41]. Moreover, metabolically healthy adipose tissue secretes anti‐inflammatory cytokines, maintains immune homeostasis, and suppresses chronic inflammation [42], which may explain why lower VAI levels do not significantly increase mortality risk. However, when log_2_‐VAI exceeds 1.81, adipose tissue storage capacity becomes compromised, leading to increased FFA release and deposition in the liver and pancreas, thereby promoting insulin resistance and lipotoxic damage [43]. Concurrently, chronic low‐grade inflammation intensifies, as M1 macrophages infiltrate adipose tissue, triggering the secretion of pro‐inflammatory cytokines (TNF‐α, IL‐6, and MCP‐1) and further activating WBC and neutrophils [44]. This cascade exacerbates vascular injury, oxidative stress, and atherosclerosis, accelerating the rise in mortality risk. This threshold effect is consistent with the adipose tissue expandability limit hypothesis, which suggests that once adipose tissue reaches its storage capacity, its protective role diminishes, leading to FFA spillover and systemic metabolic dysfunction [45]. The findings of this study align with those of Wang et al., who proposed a VAI minimum risk point of 1.7; however, their study did not quantitatively validate its statistical significance or underlying mechanisms [5]. Our study not only confirmed the statistical significance of this threshold but also elucidated the central role of inflammation and metabolic dysfunction through mediation analysis, indicating that below 1.81, metabolic function remains adaptable, whereas above this threshold, systemic inflammation and metabolic dysregulation become dominant factors, significantly increasing mortality risk.

Subgroup analysis in this study indicated that the link between log_2_‐VAI and mortality risk was more pronounced in middle‐aged individuals (40–60 years) and those without CHD. This finding may reflect the fact that middle‐aged individuals are at a critical transitional stage for metabolic and inflammatory responses, where lifestyle factors combined with metabolic burdens make them more susceptible to the impact of high VAI levels. In contrast, no significant association was observed in individuals aged < 40 years, likely due to their lower baseline mortality risk and overall better health status, which reduce the relative contribution of VAI to mortality risk. Furthermore, metabolic disorders and chronic inflammatory conditions are less pronounced in this age group, which may prevent a significant increase in mortality risk. For older individuals, mortality risk is predominantly driven by comorbidities, potentially overshadowing the independent role of VAI. Similarly, in patients with CHD, premature mortality due to cardiovascular events may dilute the contribution of VAI to all‐cause mortality analysis. To more precisely quantify the role of VAI in different populations, future research could employ competing risk models to further dissect its independent contribution to all‐cause mortality.

Given the high metabolic and inflammatory risk profile of middle‐aged individuals, this study suggests that targeted health interventions for this population may significantly reduce mortality risk. At the individual level, adherence to a Mediterranean diet may help reduce pro‐inflammatory cytokine secretion, while regular moderate‐intensity aerobic exercise (e.g., brisk walking or jogging) can improve metabolic function and lower VAI. At the public health level, integrating VAI assessment into routine health check‐ups, chronic disease management programs, and community healthcare services is recommended to optimize personalized health management strategies. Additionally, community‐based health promotion programs could include dietary counseling at community health centers to develop anti‐inflammatory dietary plans and regular monitoring of WBC and neutrophil counts, with personalized health reports provided accordingly.

Using NHANES data from a nationally representative sample, this study was the first to identify the pivotal mediation of inflammatory biomarkers (WBC and neutrophil counts) in linking VAI to all‐cause mortality. It also highlighted the unique characteristics of middle‐aged individuals in metabolic health management, providing new evidence for public health policies and personalized interventions. Nonetheless, this research is subject to certain limitations. First, causal relationships cannot be determined due to the limitations of the cross‐sectional design, and the lack of competing risk models may have underestimated the independent risks in specific populations. Second, the selection of inflammatory biomarkers was limited, which may not comprehensively reflect the inflammatory state. Third, we relied on the public‐use linked mortality files; because external‐cause categories are unavailable for 2015–2018, we could not uniformly exclude trauma/external‐cause deaths across all cycles. This constraint may introduce slight dilution toward the null; however, our primary inferences regarding the nonlinear pattern and the validated threshold are unlikely to be sensitive to this limitation. Furthermore, as an observational study, this research did not validate the actual effects of inflammation modulation on reducing mortality risk in individuals with high VAI through interventional trials. Additionally, although we calculated an *E*‐value to assess the potential impact of unmeasured confounders on the association between VAI and all‐cause mortality, it is important to note that the *E*‐value method does not account for all potential covariates or their interactions; therefore, residual confounding cannot be entirely ruled out. Future studies should incorporate competing risk models, a broader range of inflammatory biomarkers, and randomized controlled trials to further corroborate and extend the conclusions of this study.

## 5. Conclusions

This study identified a notable nonlinear relationship between log_2_‐VAI and all‐cause mortality and identified inflammation as a critical mediating mechanism in this relationship. Mediation investigation showed that WBC and neutrophil counts mediated 45.07% and 37.91% of the association, respectively, further emphasizing the pivotal role of systemic inflammation in the metabolic risks associated with elevated VAI. These findings provide new scientific evidence for designing inflammation‐targeted public health strategies to effectively mitigate obesity‐related mortality risks.

## Ethics Statement

The NHANES dataset used in this study is publicly available and was approved by the National Center for Health Statistics Research Ethics Review Board. All participants provided written informed consent prior to participation.

## Consent

Please see the ethics statement.

## Conflicts of Interest

The authors declare no conflicts of interest.

## Author Contributions

Conceptualization: Yanmei Yu and Tongcai Tan.

Formal analysis: Yanmei Yu, Wei Yang, and Zhitao Xu.

Funding acquisition: Yanmei Yu.

Supervision: Yong Liu and Tongcai Tan.

Writing–original draft: Yanmei Yu and Yong Liu.

Writing–review and editing: Yanmei Yu and Yong Liu.

## Funding

This study was supported by the Medical and Health Science and Technology Project of Zhejiang Province (Grant Nos. 2024KY746, 2023KY039, 2023KY032, and 2021KY507).

## Supporting Information

Additional supporting information can be found online in the Supporting Information section.

## Supporting information


**Supporting Information 1** Supporting Table S1. Mediation analysis of inflammation‐related indicators in the association between log2‐VAI and all‐cause mortality.

**Supporting Information 2** Supporting Table S2. Sex‐specific quintile cut‐off ranges of the VAI.

## Data Availability

All data in the manuscript are based on public data for secondary data analysis without ethical approval or primary data, and all data can be accessed at the NHANES database (https://wwwn.cdc.gov/nchs/nhanes/).
