# Supplementary material for: Decoding the Nonlinear Association Between Visceral Adiposity Index and All‐Cause Mortality: The Mediating Role of White Blood Cells and Neutrophils
Source: Int J Endocrinol. 2025 Dec 30;2025:3116986. doi: 10.1155/ije/3116986 (PMC12753583; doi:10.1155/ije/3116986)
Supplement: Supplementary file 1 — Supporting Information 1 Supporting Table S1. Mediation analysis of inflammation‐related indicators in the association between log2‐VAI and all‐cause mortality. [file IJE-2025-3116986-s001.doc]

**Table S1.** Mediation analysis of inflammation-related indicators in the association between log₂-VAI and all-cause mortality.

|  | **Mediation effect (95% CI), P value** | | |  |
| --- | --- | --- | --- | --- |
|  | Total effect | Indirect effect | Direct effect | Mediation |
| **All-cause mortality** |  |  |  |  |
| WBC | -3.91 (-7.86, -0.21), 0.0420 | -1.79(-2.32, -1.31), <0.0001 | -2.12 (-6.16, 1.63), 0.2980 | 45.07% |
| Neutrophil count | -4.31 (-8.25, -0.62), 0.0200 | -1.65(-2.04, -1.31), <0.0001 | -2.65 (-6.62, 1.09), 0.2040 | 37.91% |
| Lymphocyte count | -3.77 (-7.80, 0.01), 0.0520 | 0.06 (-0.45, 0.54), 0.8220 | -3.83 (-8.01, -0.00), 0.0500 | -1.79% |
| RDW | -3.46 (-7.20, 0.12), 0.0600 | 0.32 (0.10, 0.58), 0.0040 | -3.78 (-7.57, -0.19), 0.0320 | -8.84% |
| NLR | -4.84(-8.87,-1.05), 0.0100 | 0.17(0.01,0.36), 0.0480 | -5.02(-9.07,-1.22), 0.0080 | -3.48% |

Adjust for: age, gender, race, education level, income-to-poverty ratio, marital status, smoking status, alcohol use, physical activity, CHF, CHD, stroke, hypertension, diabetes and TC. Abbreviations: VAI, visceral fat index; CHF, congestive heart failure; CHD, coronary heart disease; TC, total cholesterol, WBC, white blood cell count; RDW, red blood cell distribution width; NLR, neutrophil-to-lymphocyte ratio;
