# Supplementary material for: Decoding the Nonlinear Association Between Visceral Adiposity Index and All‐Cause Mortality: The Mediating Role of White Blood Cells and Neutrophils
Source: Int J Endocrinol. 2025 Dec 30;2025:3116986. doi: 10.1155/ije/3116986 (PMC12753583; doi:10.1155/ije/3116986)
Supplement: Supplementary file 2 — Supporting Information 2 Supporting Table S2. Sex‐specific quintile cut‐off ranges of the VAI. [file IJE-2025-3116986-s002.docx]

**Table S2.** Sex-specific VAI quintile ranges.

| **Visceral Adiposity Index** | | | | | |
| --- | --- | --- | --- | --- | --- |
|  | **Q1** | **Q2** | **Q3** | **Q4** | **Q5** |
| **Male** | ＜1.758 | 1.758 - 2.738 | 2.739 - 4.107 | 4.109 - 6.494 | ＞6.494 |
| **Female** | ＜1.904 | 1.904 - 2.855 | 2.857 - 4.179 | 4.181 - 6.495 | ＞6.496 |
